# Effects of Liraglutide on Myocardial Function After Cardiac Surgery: A Secondary Analysis of the Randomised Controlled GLOBE Trial

**DOI:** 10.3390/jcm9030673

**Published:** 2020-03-02

**Authors:** Abraham H. Hulst, Maarten J. Visscher, Thomas G. V. Cherpanath, Lieke van de Wouw, Marc B. Godfried, Bram Thiel, Bastiaan M. Gerritse, Thierry V. Scohy, R. Arthur Bouwman, Mark G. A. Willemsen, Markus W. Hollmann, J. Hans DeVries, Benedikt Preckel, Jeroen Hermanides

**Affiliations:** 1 Department of Anesthesiology, Amsterdam UMC, University of Amsterdam, Meibergdreef 9, Postbus 22660, 1105 AZ Amsterdam, The Netherlands; a.h.hulst@amsterdamumc.nl (A.H.H.); m.j.visscher@amsterdamumc.nl (M.J.V.); liekevdwouw@gmail.com (L.v.d.W.); m.w.hollmann@amsterdamumc.nl (M.W.H.); j.hermanides@amsterdamumc.nl (J.H.); 2 Department of Anesthesiology, OLVG, Oosterpark 9, 1091 AC Amsterdam, The Netherlands; M.B.Godfried@olvg.nl (M.B.G.); b.thiel@olvg.nl (B.T.); 3 Department of Anesthesiology, Amphia, Molengracht 21, 4818 CK Breda, The Netherlands; BGerritse@amphia.nl (B.M.G.); TScohy@amphia.nl (T.V.S.); 4 Department of Intensive Care, Amsterdam UMC, University of Amsterdam, Meibergdreef 9, Postbus 22660, 1105 AZ Amsterdam, The Netherlands; t.g.cherpanath@amsterdamumc.nl; 5 Department of Anesthesiology, Catharina Hospital, Michelangelolaan 2, 5623 EJ Eindhoven, The Netherlands; arthur.bouwman@catharinaziekenhuis.nl (R.A.B.); mark.willemsen@catharinaziekenhuis.nl (M.G.A.W.); 6 Department of Endocrinology, Amsterdam UMC, University of Amsterdam, Meibergdreef 9, Postbus 22660, 1105 AZ Amsterdam, The Netherlands; j.h.devries@amsterdamumc.nl

**Keywords:** liraglutide, placebo, randomised controlled trial, Troponin, CK-MB, left ventricular function, echocardiography, cardiac function

## Abstract

Introduction: Previous studies demonstrated the cardioprotective properties of glucagon-like peptide-1 receptor agonists in patients with diabetes or cardiac disease. We investigated whether preoperative subcutaneous liraglutide improves myocardial function after cardiac surgery. Methods: We performed a pre-planned secondary analysis of adult patients undergoing cardiac surgery included in the GLOBE trial. Patients were randomised to receive 0.6 mg subcutaneous liraglutide on the evening before surgery and 1.2 mg after induction of anaesthesia, or matching placebo. Perioperative echocardiographic assessments, haemodynamic parameters, doses of vasoactive inotropic support and postoperative measurements of troponin, Creatine Kinase-MB , creatinine and lactate were compared between groups. Results: The study population consisted of the entire intention-to-treat cohort of the GLOBE trial. In this study, 129 patients received liraglutide and 132 patients placebo. Baseline characteristics were comparable between groups. Postoperatively, 170 (65%) patients underwent echocardiography. In the liraglutide group, more patients had a normal left ventricular systolic function (68%, 59 patients) compared to placebo (53%, 44 patients), difference = 15%, 95%CI = 0–30, *p* = 0.049. Assessment of the right ventricle revealed no difference in function. Conclusions: Patients receiving short-term preoperative liraglutide treatment better maintained normal myocardial function after cardiac surgery. This study warrants further evaluation of the potential beneficial effects of GLP-1 receptor agonists in cardiac surgery patients.

## 1. Introduction

Glucagon-like peptide-1 receptor agonists (GLP-1RA) improve perioperative glucose regulation, without increasing the incidence of hypoglycaemia compared to insulin [1]. In addition, various cardioprotective mechanisms have been attributed to GLP-1RAs. We recently reported that two preoperative subcutaneous injections of liraglutide (a long-acting GLP-1RA) improved glycaemic control during cardiac surgery (GLOBE trial) [2]. In this secondary analysis of the GLOBE trial, we aimed to evaluate the effect of liraglutide on postoperative cardiac function through analysis of postoperative echocardiography, haemodynamic parameters, and routinely collected biomarkers of cardiac injury.

GLP-1 has been reported to increase myocardial metabolic efficiency of glucose usage, reduce systemic and pulmonary vascular resistance and activate ischaemic preconditioning pathways [3,4,5]. However, these mechanisms have mainly been demonstrated in animal studies, while there are only a few physiological studies in humans [6]. Some indications of positive GLP-1 mediated effects on cardiac function were observed in clinical studies, showing an improved left ventricular function and reduced infarct size after ischaemic injury in GLP-1 treated subjects [7,8,9]. These results were mainly seen in patients with coronary artery disease undergoing dobutamine stress testing or percutaneous coronary interventions for acute myocardial infarction and were assessed at a limited interval after the intervention (up to 72 h) [6]. More recently, longer-term treatment with liraglutide was shown to reduce the incidence of major cardiovascular complications in high-risk patients with type 2 diabetes mellitus [10,11].

From the dataset of a recently reported multicentre, randomised, placebo-controlled, trial, wherein we administered a long-acting GLP-1 receptor agonist, liraglutide, to patients undergoing cardiac surgery, we here report a pre-planned secondary analysis of indicators of cardiac function collected in routine clinical care. Based on the aforementioned studies, we hypothesised that liraglutide, compared to placebo, improves postoperative cardiac function.

## 2. Materials and Methods

### 2.1. Study Design

This study is a secondary analysis of the GLOBE trial, a multicentre, triple-blind, placebo-controlled, parallel-group, phase 3, randomised superiority clinical trial which ran in four Dutch tertiary care centres. The primary hypothesis of the GLOBE trial was that the preoperative administration of liraglutide reduces the number of patients requiring insulin for glycaemic control during cardiac surgery. The trial was registered with www.trialregister.nl, number NTR6323. The study protocol was approved by the medical ethics committee of the Amsterdam UMC (registration number: 2017_012) before initiation of the trial. The detailed study protocol is available open access [12], and the primary results of the GLOBE trial have recently been published [2]. The relevant methodology concerning this cardiac analysis is described below. We wrote this paper in adherence to the CONSORT recommendations for reporting of randomised trials [13].

### 2.2. Participants

Patients planned to undergo elective cardiac surgery aged between 18 and 80 years were eligible for inclusion. We excluded patients with type 1 diabetes, current treatment with insulin >0.5 IU/kg daily, GLP-1 RAs, or corticosteroids, history of heart failure (New York Heart Association [NYHA] class III and IV) [on 6 November 2017, this was amended to NYHA class IV only, after an update in the summary of products characteristics of liraglutide]), impaired renal function (creatinine ≥133 μmol/L for men and ≥115 μmol/L for women), allergies to trial products, history of pancreatic surgery, acute or chronic pancreatitis, personal or family history of medullary thyroid cancer or multiple endocrine neoplasia syndrome type 2, and (possibly) pregnant or breastfeeding women. All participants had to provide written informed consent before any trial-related procedures.

### 2.3. Randomisation and Masking

At each institution, central research pharmacists allocated patients after on-line randomisation through an electronic data management system. Randomisation was balanced (1:1), with variable random blocks of four, six, or eight patients, and stratified per centre and type 2 diabetes mellitus. The research pharmacy distributed study medication in visually identical pen-injectors, equal in appearance and weight (provided by Novo Nordisk) directly to trained research personnel, responsible for the administration of the study medication. All patients, care providers, and study personnel were thus blinded to treatment allocation.

### 2.4. Procedures

Patients received a first subcutaneous injection with liraglutide 0.6 mg (Novo Nordisk A/S, Bagsvaerd, Denmark) or placebo on the evening before surgery (after 15:00 h) and a second dose of 1.2 mg or placebo was given after the induction of anaesthesia. Starting with the induction of anaesthesia and lasting until transfer to the intensive care unit (ICU), researchers measured blood glucose concentrations every hour; an intravenous insulin bolus injection algorithm was used for targeting intra-operative blood glucose concentrations between 4.0–8.0 mmol/L [12]. After transfer to the ICU, study interventions stopped, and further treatment, including blood glucose management, was left to the discretion of the ICU physician.

### 2.5. Data collection and Outcomes

Baseline characteristics, comorbidities, perioperative haemodynamic data and glycaemic control were recorded per study protocol as reported previously [2]. Echocardiography was performed as part of routine perioperative care by the treating cardiologist. We collected transthoracic echocardiographic assessments before, and up to thirty days after surgery. In case of multiple investigations, we recorded the assessment closest to the day of surgery. We recorded qualitative assessment (categorised as normal, or mildly, moderately or severely reduced function) of right and left ventricular function as noted by the echocardiographer. We recorded heart rate, heart rhythm and mean arterial pressure from the continuous recordings stored in the patient electronic health records from the start of surgery until 24 h after surgery, or discharge of the patients from the ICU, whichever occurred first. Data were recorded at predefined time-points; at start of surgery, end of surgery, and 1, 6, 12, and 18 h thereafter. Noted measurements were the means of the three values before, at, and after the respective time points. From the ICU electronic health records, we also noted the total dose of norepinephrine, dobutamine, milrinone, and amiodarone administered in the first 48 postoperative hours. In all participating centres, as part of routine clinical care, either Creatine Kinase-MB (CK-MB) or Troponin T levels were recorded postoperatively until two consecutive measurements showed a decline in these markers of cardiac injury. Hence, periods between these measurements varied, and we, therefore, analysed peak postoperative values in the first 24 h. We also noted lactate levels in this period, and creatinine measurements obtained up to five days after surgery.

### 2.6. Statistical Analysis

The sample size was defined by the number of patients included in the intention to treat analysis of the GLOBE trial [2]. Discrete data are presented as count (%) and compared between groups using χ² tests or Fisher’s exact test. Continuous variables are presented as mean (SD) or median (IQR) and compared using Student’s *t*-test or Mann-Whitney U tests, depending on the distribution of the data. Absolute differences between groups are presented with the respective 95% CIs. Normality of distributions was assessed visually with histograms, Q-Q plots, and the Shapiro-Wilk test. All statistical tests were 2-sided, and a *p* value of less than 0.05 was considered significant. Statistical analyses were performed using IBM SPSS Statistics (version 26, IBM Corp., Armonk, N.Y., USA).

### 2.7. Role of the Funding Source

The funder of the study had no role in study design, data collection, data analysis, data interpretation, or writing of the report. The corresponding author had full access to all the data in the study and had final responsibility for the decision to submit.

## 3. Results

The cohort of patients consists of all 261 patients included in the primary intention-to-treat analysis of the GLOBE trial. Of these, 129 patients were allocated to the liraglutide group and 132 to the placebo group. Baseline characteristics were well balanced and are summarised in Table 1. In this trial we observed that patients treated with liraglutide required less insulin for glycaemic control during surgery, compared to placebo, and also had lower glucose concentrations during surgery and ICU admittance [2].

### 3.1. Echocardiography

**Figure 1:**
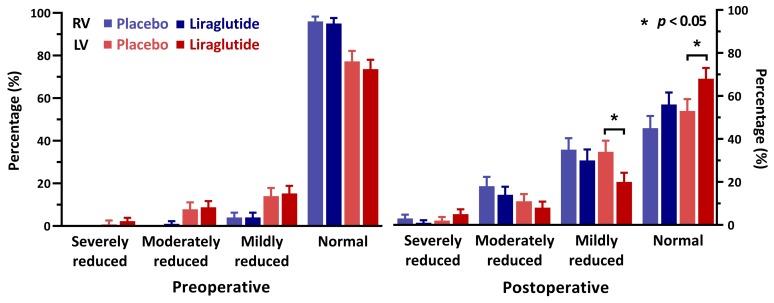
Echocardiographic assessment of cardiac function before and after surgery, percentage of patients. RV = right ventricle, LV = left ventricle.

Preoperative echocardiographic assessment of left ventricular function was reported for all included patients; however, postoperatively echocardiography was available in only 65% (170) of patients. Echocardiography was performed at a median of 4 days (IQR 3-5) after surgery. Baseline characteristics of the cohorts with and without an available echocardiographic assessment revealed no significant differences, except for the type of surgery; patients without postoperative echocardiography underwent coronary artery bypass graft surgery (CABG)-only procedures in 77% of cases, while this procedure-type comprised only 20% of the cohort that had an echocardiography postoperatively (Supplementary Material). Assessment of right and left ventricular function preoperatively and within 30 days after surgery are visualised in Figure 1. While left ventricular systolic function was comparable between groups preoperatively, we observed a higher rate of patients with a normal left ventricular systolic function in the liraglutide group compared to the placebo group (liraglutide: 59 patients (68%) vs placebo: 44 patients (53%), difference = 15% (95% CI 0–30, *p* = 0.049)). We observed no difference in right ventricular function.

### 3.2. Haemodynamics

**Figure 2:**
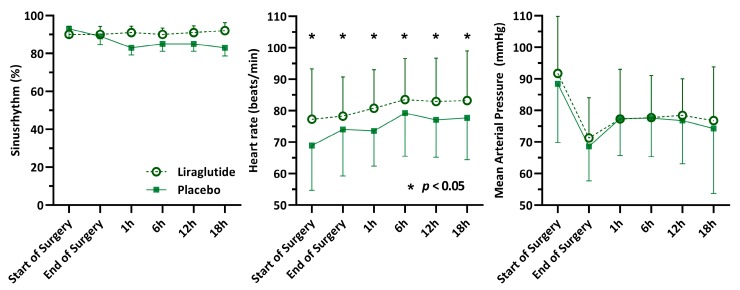
Perioperative haemodynamic measurements.

Continuous measurement of heart rate, heart rhythm, and mean arterial pressure from the start of surgery for up to 18 h after surgery were available for 81% (212) of patients (Figure 2). Mean postoperative heart rate was significantly higher in the liraglutide group, with a heart rate of 83 (±11) beats/min compared to 77 (±11) in the placebo group, (difference = 6; 95% CI 3–8, *p* < 0.001). There was no difference in mean arterial pressure at any of the time points. At every postoperative time point, most patients had sinus rhythm (>83%) without statistically significant differences between the groups (Figure 2). On the ICU, 74% (192) of patients received norepinephrine, 7% (19) dobutamine, and 7% (18) milrinone. The number of patients receiving vasoactive/inotropic support and the respective doses of different drugs did not differ between groups (Supplementary Material).

### 3.3. Biomarkers

**Figure 3:**
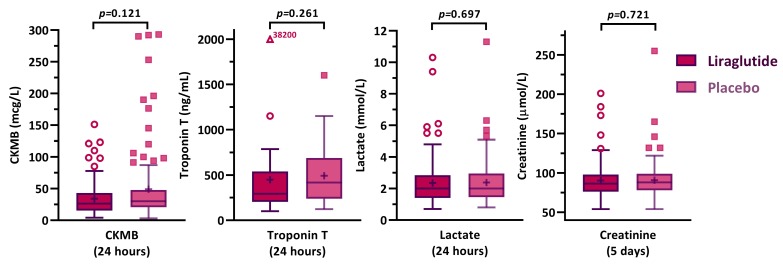
Postoperatively measured biomarkers, peak concentrations.

To monitor postoperative myocardial ischaemia, one of the four involved centres used troponin measurements (48 patients) while the other three centres measured CK-MB postoperatively (213 patients). Peak values of both markers revealed no significant differences between the liraglutide and placebo group (Figure 3). Likewise, we found no between-group difference in peak and mean lactate and creatinine levels (Supplementary Material).

## 4. Discussion

This analysis of cardiac outcomes of a randomised controlled trial suggests that liraglutide might better preserve myocardial function after cardiac surgery. Echocardiographic assessments revealed that more patients had a normal left ventricular systolic function when treated preoperatively with liraglutide, compared to patients in the placebo group in which more patients had a reduced cardiac function postoperatively. In addition, we observed an increased heart rate in liraglutide treated patients, but no differences in mean arterial pressure. Other markers of short-term cardiac function such as vasoactive/inotropic support or levels of biomarkers seemed unaffected by preoperative liraglutide treatment.

Studies on GLP-1 induced cardioprotection are limited, and all used different combinations of patient populations (with variability in cardiovascular health), incretin interventions (with differing GLP-1 receptor agonistic mechanisms) and cardiac outcome (using various imaging techniques and biomarkers) [6]. Our current study differs from previous studies on most of these aspects. Many previous studies analysed patients with ischaemia-induced cardiac injury after percutaneous coronary interventions. GLP-1 infusion improved left ventricular ejection fraction determined by echocardiography following successful percutaneous coronary intervention (PCI) [7], and it reduced left ventricular dysfunction after balloon occlusion [8]. In a later study, a six-hour exenatide infusion after PCI reduced infarct size on cardiac magnetic resonance imaging [14]. Also, twice daily exenatide (a GLP-1 receptor agonist) for 72 h reduced infarct size on cardiac magnetic resonance imaging and cardiac biomarker release [15]. However, both studies observed no functional differences in echocardiography [14,15]. While we did find a better echocardiographic function in the liraglutide group, compared to placebo, our population consisted of patients undergoing cardiac surgery, in which the release of biomarkers is caused mostly by direct surgical injury to the cardiac muscle, contrasted by ischaemia-induced release during PCI. In addition, biomarker release varies according to the magnitude and number of surgical interventions. Based on the measurement of biomarkers of cardiac injury, we detected no evidence of cardioprotection in our study. Unfortunately, other techniques used before, such as cardiac magnetic resonance imaging, were unavailable in our patient population due to the reliance on routine clinical diagnostics for the outcome of this sub-study.

Another difference with previous studies is the type of GLP-1 RA studied. While we performed the first randomised trial using liraglutide in patients undergoing cardiac surgery [2], Besch et al. randomised patients undergoing CABG to either a continuous exenatide infusion or insulin for glycaemic control [16]. Similar to our present study, the authors performed an analysis of cardiac outcomes and found no difference in postoperative troponin levels nor in the incidence of reduced left ventricular ejection fraction between treatment and control groups [17]. Of note, the primary outcome of this trial (time spent within the glycaemic target range) also did not reach a significant difference between groups [16]. In contrast, liraglutide proved effective in improving glycaemic control during cardiac surgery in our patient population [2] and the current analysis also reveals a signal of improved cardiac outcomes.

Indications of beneficial effects on cardiac outcomes are not only based on preclinical data and the aforementioned studies of ischaemia-induced cardiac injury. Currently, indications of GLP-1 mediated cardioprotection are reinforced by the results from large cardiovascular outcome trials in patients with diabetes mellitus. In the LEADER trial, patients with diabetes mellitus randomised to receive liraglutide had lower rates of major adverse cardiovascular events, including cardiovascular death, compared to placebo [10]. Similarly, dulaglutide, another long-acting GLP-1 RA, reduced the composite incidence of myocardial infarction, stroke and cardiovascular death [11]. Haemodynamically, both trials observed an increase in heart rate and a reduction in mean arterial pressure in the intervention group. In the present study, the most statistically robust findings were also the higher heart rates observed in the liraglutide group. This effect has been consistently demonstrated in previous studies [16,17,18,19] and GLP-1 receptors have been found in the sinoatrial node [10,20,21]. Some authors have found GLP-1 RAs to induce vasodilation and microvascular recruitment, resulting in lower systemic vascular resistance [21] as well as an atrial natriuretic peptide-mediated reduction of systolic and diastolic blood pressures [4]. Although we found no differences in mean arterial pressure (with a concurrent increase in heart rate in the liraglutide group), the available data are insufficient to infer whether this is due to a reduction in systemic vascular resistance, cardiac preload or contractility.

### Limitations

This study has some limitations: it is a secondary analysis of a randomised clinical trial. As such, results should be interpreted cautiously and only as supportive evidence of the hypothesis that GLP-1RA might improve cardiovascular outcomes after cardiac surgery. Secondly, outcomes were collected from echocardiographic, haemodynamic and laboratory data coming from routine clinical care, and therefore some parameters suffered from missing data. Specifically, follow-up of valve surgery more often included echocardiography, compared to CABG-only procedures. However, the cohorts of patients with and without postoperative echocardiography had comparable baseline characteristics. Thus, we deemed it unlikely that a significant bias influenced the decision of whether or not postoperative echocardiography was performed. Furthermore, while an echocardiographic study was performed in most patients postoperatively, only qualitative assessments of cardiac function were consistently reported, and more quantitative measurements were not available.

In conclusion, liraglutide administered before cardiac surgery modestly improved postoperative cardiac function. It altered immediate haemodynamics (increased heart rate) and better preserved left ventricular function on echocardiography at postoperative follow-up. This warrants further investigation of liraglutide in larger trials of cardiac surgery patients with a primary focus on postoperative cardiovascular outcomes.

## Figures and Tables

**Table 1:** Baseline characteristics of the intention-to-treat population.

			All	Liraglutide	Placebo
			261	129	132
Age, mean (SD), years			65.0 (10.9)	64.6 (11.2)	65.3 (10.7)
Male sex, No. (%)			211 (81)	105 (81)	106 (80)
ASA score III, No. (%)			189 (72)	94 (73)	95 (72)
Smoker past year, No. (%)			54 (21)	26 (20)	28 (21)
Hypertension, No. (%)			111 (43)	57 (44)	54 (43)
BMI, mean (SD), kg/m^2^			27.5 (4.2)	27.3 (4.0)	27.7 (4.4)
Diabetes mellitus type 2, No. (%)			42 (16)	21 (16)	21 (16)
Creatinine clearance, mean (SD), mL/min			80.4 (16.6)	80.6 (17.0)	80.2 (16.2)
Glycated haemoglobin, mean (SD), mmol/mol			40 (8.9)	40 (9.7)	40 (8.1)
EuroSCORE II, median (IQR), %			1.27 (0.89–1.97)	1.22 (0.84–1.93)	1.34 (0.90–2.05)
Left ventricular function < 50%, No. (%)			64 (25)	34 (26)	30 (23)
Type of surgery, No. (%)					
	CABG-only procedure		92 (35)	46 (36)	46 (35)
	Single non-CABG procedure		102 (39)	52 (40)	50 (38)
	Combined procedures		67 (26)	31 (24)	36 (27)
Duration of surgery, median (IQR), min			222 (165–293)	222 (162–276)	219 (169–308)
Type of anaesthesia maintenance, No. (%)					
	Propofol		16 (6)	8 (6)	8 (6)
	Sevoflurane		245 (94)	121 (94)	124 (94)

There were no significant differences between the two treatment groups for any of the baseline characteristics. ASA = American Society of Anesthesiologists, CABG = Coronary artery bypass surgery.

## Supplementary Materials

The following are available online at https://www.mdpi.com/2077-0383/9/3/673/s1.
