# Supplementary material for: Effects of Liraglutide on Myocardial Function After Cardiac Surgery: A Secondary Analysis of the Randomised Controlled GLOBE Trial
Source: J Clin Med. 2020 Mar 2;9(3):673. doi: 10.3390/jcm9030673 (PMC7141279; doi:10.3390/jcm9030673)
Supplement: Supplementary file 1 [file jcm-09-00673-s001.zip › Supplementary Material/Contributions to the GLOBE study group.docx]

**Contributions to the GLOBE study group**

**Author group**

Abraham H. Hulst MD^1,2,3^, Maarten J. Visscher MD^1^, Marc B Godfried PhD^2^, Bram Thiel^2^, Bastiaan M Gerritse PhD^3^, Thierry Scohy PhD^3^, R Arthur Bouwman PhD^4^, Mark G A Willemsen MD^4^, Markus W. Hollmann PhD^1^, Benedikt Preckel PhD^1^, J. Hans DeVries PhD^5^, Jeroen Hermanides PhD^1^

**Affiliations of author group members**

^1^ Department of Anaesthesiology, Amsterdam UMC, University of Amsterdam, Meibergdreef 9, Postbus 22660, 1105 AZ Amsterdam, the Netherlands

^2^ Department of Anaesthesiology, OLVG, Oosterpark 9, 1091 AC Amsterdam, the Netherlands.

^3^ Department of Anaesthesiology, Amphia, Molengracht 21, 4818 CK Breda, the Netherlands.

^4^ Department of Anaesthesiology, Catharina Ziekenhuis, Michelangelolaan 2, 5623 EJ Eindhoven, the Netherlands.

^5^ Department of Internal Medicine, Amsterdam UMC, University of Amsterdam, Meibergdreef 9, Postbus 22660, 1105 AZ Amsterdam, the Netherlands

**Contributions of author group members**

Abraham Hulst was coordinating investigator, was involved in the design of the trial, collected, verified, and analysed data and drafted this report. Maarten Visscher was involved in data collection, verification and analysis. Marc Godfried, Bram Thiel, Bas Gerritse, Thierry Scohy, Arthur Bouwman, and Mark Willemsen were involved in data collection and verification. Markus W. Hollmann and Benedikt Preckel were involved in the design of the trial. Hans DeVries was involved in the design of the trial, data analysis and drafting the manuscript. Jeroen Hermanides was principal investigator, was involved in the design of the trial, data analysis and drafted this report. All members of the author group listed here have commented on the analyses and drafts of this report and have seen and approved the final version of the report.

**Acknowledgements**

We would like to acknowledge all the patients and their families who participated in the GLOBE trial. As well as all anaesthetists, nurse anaesthetists and perfusionists who assisted with blood glucose measurements and insulin administration, without whom the trial would not have been possible.

**Participating centres**

We have listed each hospital with the total number of patients recruited in [n], followed by the names of the local principal investigator(s), and other significant contributors in that centre. The hospitals are ordered depending on the numbers recruited.

**Amsterdam UMC, University of Amsterdam, Amsterdam [104]**Jeroen Hermanides (PI), Abraham H Hulst, Maarten J Visscher, Markus W Hollmann, Benedikt Preckel, J Hans DeVries, Jorinde A W Polderman, Thomas G V Cherpanath, Marije Schipper, Pieter Raps, Rowan van der Peet, Valerie Geukes, Lieke van de Wouw, Susanne Eberl, Antoine H G Driessen

**OLVG locatie oost, Amsterdam [73]**

Marc B Godfried (PI), Bram Thiel, Rosa van Dorst, Felicia van Schie, Ricardo Cocchieri, Peter HJ van der Voort

**Amphia, Breda [48]**

Bastiaan M Gerritse (PI) Thierry Scohy, Nardo J van der Meer, Pim van der Heiden

**Catharina Ziekenhuis, Eindhoven [36]**

R Arthur Bouwman (PI), Mark G A Willemsen, Marc P Buise, Joris van Houte

**Email addresses**

Jeroen Hermanides [j.hermanides@amc.uva.nl](mailto:j.hermanides@amc.uva.nl)

Abraham Hulst [a.h.hulst@amc.uva.nl](mailto:a.h.hulst@amc.uva.nl)

Maarten Visscher [m.j.visscher@amc.uva.nl](mailto:m.j.visscher@amc.uva.nl)

Markus Hollmann [m.w.hollmann@amc.uva.nl](mailto:m.w.hollmann@amc.uva.nl)

Benedikt Preckel [b.preckel@amc.uva.nl](mailto:b.preckel@amc.uva.nl)

Hans de Vries [j.h.devries@amc.uva.nl](mailto:j.h.devries@amc.uva.nl)

Jorinde Polderman [j.a.polderman@amc.uva.nl](mailto:j.a.polderman@amc.uva.nl)

Thomas Cherpanath [t.g.cherpanath@amc.uva.nl](mailto:t.g.cherpanath@amc.uva.nl)

Marije Schipper [m.c.schipper@amc.uva.nl](mailto:m.c.schipper@amc.uva.nl)

Pieter Raps [pieter_raps@hotmail.com](mailto:pieter_raps@hotmail.com)

Rowan van der Peet [rowanvanderpeet@hotmail.com](mailto:rowanvanderpeet@hotmail.com)

Valerie Geukes [v.d.geukes@student.vu.nl](mailto:v.d.geukes@student.vu.nl)

Lieke van de Wouw [L.vandewouw@amc.uva.nl](mailto:L.vandewouw@amc.uva.nl)

Suzanne Eberl [s.eberl@amc.uva.nl](mailto:s.eberl@amc.uva.nl)

Antoine Driessen [a.h.driessen@amc.uva.nl](mailto:a.h.driessen@amc.uva.nl)

Marc Godfried [M.B.Godfried@olvg.nl](mailto:M.B.Godfried@olvg.nl)

Bram Thiel [b.thiel@olvg.nl](mailto:b.thiel@olvg.nl)

Rosa van Dorst [rosavandorst@hotmail.com](mailto:rosavandorst@hotmail.com)

Felicia van Schie [f.m.vanschie@amc.uva.nl](mailto:f.m.vanschie@amc.uva.nl)

Ricardo Cocchieri

Peter HJ van der Voort

Bastiaan Gerritse [BGerritse@amphia.nl](mailto:BGerritse@amphia.nl)

Thierry Scohy [TScohy@amphia.nl](mailto:TScohy@amphia.nl)

Nardo J van der Meer

Pim van der Heiden

Arthur Bouwman [arthur.bouwman@catharinaziekenhuis.nl](mailto:arthur.bouwman@catharinaziekenhuis.nl)

Mark Willemsen [mark.willemsen@catharinaziekenhuis.nl](mailto:mark.willemsen@catharinaziekenhuis.nl)

Marc P Buise

Joris van Houte
